# *Oportunidades* Program Participation and Body Mass Index, Blood Pressure, and Self-Reported Health in Mexican Adults

**Published:** 2008-06-15

**Authors:** Lia C H Fernald, Xiaohui Hou, Paul J Gertler

**Affiliations:** School of Public Health, University of California, Berkeley; The World Bank, Washington, DC; University of California, Berkeley, Berkeley, California; Instituto Nacional de Salud Pública, Cuernavaca, Mexico

## Abstract

**Introduction:**

Governments around the world are seeking to address the increasing prevalence of obesity and hypertension. Our objective was to evaluate the effect of an incentive-based development program (*Oportunidades*, formerly *Progresa*) on body mass index (BMI), blood pressure, and self-reported health.

**Methods:**

An intervention group of low-income (below the 20th percentile nationally), rural, Mexican adults (aged 30–65 years) (n = 5280) received program benefits (cash transfers contingent on positive changes in health behavior such as regular health checkups) for 3.5 to 5.0 years. They were compared with a newly recruited control group of adults (n = 1063) who had not yet begun receiving benefits. Analyses were adjusted for almost 50 social and economic covariates.

**Results:**

Age- and sex-adjusted BMI was lower in adults from intervention communities than in those from control communities (26.57 kg/m^2^ vs 27.16 kg/m^2^, *P* < .001), as was the prevalence of obesity (20.28% vs 25.31%, *P* < .001) and overweight (59.24% vs 63.04%, *P* = .03); these results were attenuated after covariates were included. Adults in intervention communities had a lower combined prevalence of uncontrolled hypertension (33.80% vs 34.52%, *P* = .008) when adjusting for all covariates. Mean systolic (β = –2.60, *P *< .001) and diastolic (β = –2.84, *P* < .001) blood pressures were significantly lower in the intervention communities after all covariates were included, and self-reported health outcomes were better.

**Conclusion:**

Participation in *Oportunidades*, a large-scale cash-transfer program, was associated with lower prevalence of obesity and hypertension and better self-reported health in adults in rural Mexico.

## Introduction

Many developing countries are starting to parallel the developed world in terms of an increasing prevalence of obesity, which is one of the primary risk factors for noncommunicable chronic diseases such as hypertension (1). Obesity increases the risk of dyslipidemia, hypertension, hyperinsulinemia, insulin resistance, and diabetes, all of which substantially increase the risk for cardiovascular disease (2). In addition, obese persons are at higher risk for gallbladder disease, certain types of cancer, sleep apnea, respiratory problems, osteoarthritis, emotional distress, discrimination, and social stigmatization (3). In women, obesity is associated with disturbances in the reproductive system, such as menstrual cycle disturbances, infertility, and polycystic ovary syndrome (4). In 2001, chronic disease contributed to approximately 60% of the 56.5 million total reported deaths in the world and to approximately 46% of the global prevalence of disease (5).

The World Health Organization has issued a call to action to put overweight and obesity at the forefront of public health policies and programs (6). Some governments in the developing world have launched programs that focus on preventing obesity and chronic diseases (7). For example, *Agita* in Brazil has increased awareness about the importance of physical activity (8,9), but no evidence has shown that the program changed obesity rates. Other community-based programs include *Muévete Bogatá* in Colombia and *Vida* in Chile, in addition to programs in China, Cuba, South Korea, and Mauritius (7,10). In spite of this interest in obesity prevention, many programs have shown no improvement — or in some cases have shown an increase in body mass index (BMI) — during the intervention. Community-based programs in developing countries have had more success addressing hypertension and blood pressure than in addressing obesity (10,11).

Clear operational challenges exist to addressing the problem of obesity and hypertension in the developing world, one of which is the lack of financing and institutional capacity to approach these problems (12). The estimated total cost attributable to hypertension in Mexico, for example, was approximately $2.49 billion (in U.S. dollars) in 2007 (13). Another challenge is that the public sector in developing countries is often primarily focused on addressing the challenge and threat of communicable diseases. In many cases, health systems are set up to address acute conditions and are unable to deal with the complexity of chronic conditions over the life cycle.

Mexico has a rapidly growing prevalence of obesity and hypertension that reflects a trend in Latin America (14). The prevalence of overweight or obesity is more than 60% in women and 50% in men, even in very poor rural populations in Mexico (15). Mexico also has a high prevalence of diabetes (16), hypertension (14), dyslipidemia (17), and other risk factors for cardiovascular disease, including tobacco use (18).

In this article, we report the effect of an incentive-based poverty alleviation program, *Oportunidades* (previously *Progresa*), on BMI, blood pressure, and select health behaviors in adults. The program was originally designed to improve health and development in children and has achieved this goal in the short run, as evidenced by reductions in prevalence of stunting and anemia in preschool children (19,20). A secondary objective of the program was to improve adult health, although no analyses have thus far examined this objective. Data from the National Nutrition Survey in Mexico show that the prevalence of overweight and obesity decreases with increasing levels of socioeconomic status (21), and this trend is reflected in other countries at the same level of economic development as Mexico. In spite of this association at the national level, analyses of a low-income population in Mexico suggest that BMI and systolic blood pressure (SBP) are positively associated with socioeconomic status (22). Thus, the increased household income resulting from *Oportunidades* could contribute to an increase in the prevalence of overweight, obesity, and hypertension. However, we hypothesized that the program participation requirements (e.g., regular visits to a physician, information sessions about noncommunicable diseases) would counterbalance potential income effects, resulting in a net positive effect on BMI and blood pressure. Given the contact with health professionals that is a required component of program participation, we hypothesized that self-reported health outcomes (e.g., sick days, ability to participate in activities of daily living [ADL]) would also improve.

## Methods

### Intervention

*Oportunidades* began in 1997 as a national program designed to relieve extreme poverty in Mexico. The *Oportunidades* program combines a cash-transfer program with financial incentives for positive behavior in health, education, and nutrition. During its first 3 years, *Oportunidades* extended benefits to many eligible families in rural areas. Starting in 2001, the program expanded to urban areas. By 2004, *Oportunidades* covered approximately 5 million families in all 31 states of Mexico; it has been used as a model for initiatives in Argentina, Brazil, Colombia, Ecuador, Honduras, Jamaica, Nicaragua, Peru, Turkey, and the United States.

At its inception, *Oportunidades* determined household eligibility in 2 stages, first by identifying low-income communities and then by choosing low-income households within those communities (23). Low-income communities were selected on the basis of the proportion of households in those communities living in poverty, according to data from the 1995 national census. Households were selected according to an index of objective characteristics, such as housing materials, water and sanitation facilities, education, and family structure, which were shown to be good proxies for annual income. On average, 78% of the households in selected communities were eligible for program benefits, and 97% of these households enrolled in the program (24). Once enrolled, households received benefits for a minimum of 3 years, conditional on meeting the program requirements; new households were not able to enroll until the next certification period.

Program benefits were distributed only if family members complied with a series of behavioral changes. The requirements included prenatal care; well-baby care and immunization; nutrition monitoring and supplementation; preventive checkups; and participation in educational programs on health, hygiene, and nutrition. Adult family members were required to attend a biannual health checkup and were encouraged to participate in regular educational sessions at which health, hygiene, and nutrition issues and best practices were discussed. *Oportunidades* verified that households completed the required health care visits by having medical providers at participating public health clinics provide certification of participation.

*Oportunidades* families received 2 types of cash transfers every month. The first was a universal cash amount for all families, typically worth approximately 20%–30% of household income, equivalent to an average of approximately $25 per month. The second cash gift was given to households with school-aged children if the children were enrolled in and attended school. This amount varied depending on the number of children attending school and was greater for girls than for boys. Approximately 1% of households were denied the cash transfer because of noncompliance.

### Selection of comparison group

At the inception of *Oportunidades*, and for the purposes of conducting a rigorous evaluation of the program, eligible communities in rural areas of 7 states were randomly assigned to intervention and control groups. A baseline survey was conducted in all households in these groups in 1997 and 1998, which included information about socioeconomic status and household demographic composition but no information about anthropometry or health status. However, because the original control group was incorporated into the program 18 months after the program began, both the control and intervention groups were consolidated into one intervention group.

In 2003, a new control group was added as part of the 5-year follow-up survey of the original communities. This new group consisted of 151 control communities selected from the original 7 evaluation states. Data from the 2000 census were used to select new control communities that matched the old ones as closely as possible. Specifically, communities were selected that had not yet been incorporated into *Oportunidades* and that most closely matched the originals by propensity score matching methods applied to sociodemographic and infrastructure characteristics (P. Todd, unpublished data, 2004). Matching by using the propensity score is equivalent to matching intervention and control observations on the basis of a large number of characteristics (25). After completing the propensity score matching, we then chose the nearest community to each original community, in terms of propensity score, to be included in the comparison group. A proportion of the households in the new comparison communities was eventually invited to participate in *Oportunidades* in 2005 when the program was expanded to all rural areas. Thus, to reduce bias, we restricted the sample for analysis to just those families who were invited to participate in *Oportunidades* and subsequently enrolled in the program.

### Sample

The survey reported here was conducted in 2003 in low-income households (income below the 20th percentile, mean daily per capita expenditure of less than $2) from 323 rural communities (defined as towns of <2500 inhabitants) in 7 Mexican states. The households included in the sample were a mix of those recruited in 1997 as part of the original sample and those from new control communities first surveyed in 2003. The final sample used for analysis consisted of an intervention group of 5280 adults from households living in the original communities recruited in 1997 and 1998 and a control group of 1063 adults living in the new comparison areas at the time of the 2003 survey (these 1063 adults were subsequently enrolled in *Oportunidades* in 2005).

### Data collection and measures

Data were collected during house-to-house interviews with all participants. The interviews occurred on all days of the week except Sundays between 8 am and 6 pm. Interview teams visited the homes without appointments and returned to each home at least 3 times to try to locate household members. After identifying the head of household — or spouse of the head of household if the head of household was not available — the interviewers obtained written consent to conduct the medical assessment and the interview. The interviewers then measured and weighed each available adult and measured blood pressure. During this same visit, a questionnaire was administered to obtain information about demographic, socioeconomic, and other factors.

**BMI and obesity**

Height and weight were measured during the interview by trained personnel in duplicate by using standard techniques (26). If the two measurements differed by more than 5% for any outcome, survey personnel obtained a third measure and used the two closest measurements. Weight was measured in light clothing without shoes to the nearest 100 g on a digital scale (Tanita Mother-Baby scale, model 1582, Tanita Corp, Arlington Heights, Illinois). Height was measured in standard position with a portable stadiometer (Road Rod, model 214, seca corp, Hanover, Maryland) and recorded to the nearest millimeter. Obesity was defined as BMI ≥30.0 kg/m^2^, and overweight was defined as BMI ≥25.0 kg/m^2^.

**Blood pressure and hypertension**

Blood pressure was measured by trained nurses with mercury sphygmomanometers. Uncontrolled hypertension was defined as SBP ≥140 mm Hg or diastolic blood pressure (DBP) ≥90 mm Hg (27). Participants were also asked about symptoms of hypertension (e.g., headaches, dizziness, buzzing in the ears, seeing lights without apparent reason, nosebleeds without apparent reason).

**Self-reported health**

Questionnaires were administered by survey personnel to obtain information about self-reported health status, self-reported fitness, ability to participate in ADL, and socioeconomic status. Specifically, participants were asked if they could participate in medium-effort ADL, such as working on a farm or in a garden or sweeping. They were also asked if they were able to participate in heavy-effort ADL, such as running or lifting a heavy object. Participants were asked how many sick days they had had in the previous 4 weeks and were also asked the number of days of "inability" (not being able to perform daily activities) they had experienced in the past 4 weeks.

**Demographic and other household-level control variables**

The following individual- and household-level variables were obtained through the household questionnaire: age, sex, educational attainment and occupational status of all household members, whether head of household was married, whether head of household spoke an indigenous language, whether any household member was self-described as disabled, land use (whether a household owned and used any piece of land), ownership of farm animals, ownership of an animal other than a farm animal, amount of land owned by the household, presence of dirt floor, presence of bathroom, presence of electricity, number of large assets (including television, washing machine, gas heater, and refrigerator), number of small assets (including blender, electric kettle, radio, stereo, video cassette recorder, and fan), and ownership of car or other vehicles.

An additional questionnaire was applied to the new comparison households, which asked families retrospectively about household demographic structure and ownership of assets in 1997 and 1998. The goal of the retrospective survey was to collect information that could be easily and accurately recalled and could be incorporated into the analysis. This section of the questionnaire was extensively pilot-tested to ensure that adults in households from comparison communities could report on their household-level socioeconomic and demographic structure from 5 years before. Only questions for which survey respondents were confident in their accuracy and recall were included in the final questionnaire.

**Community-level covariates**

A community survey was also administered to obtain detailed information about community characteristics. These questions included the following variables: proportion of the population that was indigenous, proportion of the village supplied with electricity, proportion of the village with a drainage system, availability of a public or private telephone, availability of a preschool, availability of a health center, presence of a shop that serves the local community, presence of a home-based shop, whether the community received government assistance, presence of a formal or informal credit institution, presence of a high school, average rent for a house, presence of community irrigation, and mean monthly wages for men and women.

As mentioned above, for inclusion into the model, we focused principally on data obtained in the baseline survey from 1997. However, because the 1997 data for some community characteristics were unavailable, we used 2003 information as necessary.

### Statistical analyses

We estimated the impact of *Oportunidades* on adult health, focusing on 3 primary sets of outcomes: 1) BMI, overweight, and obesity; 2) blood pressure and hypertension; and 3) self-reported health and fitness. The 2 methods of analysis were ordinary least squares (OLS) regression adjusted for survey design and nonparametric matching techniques. All analyses were conducted by using STATA version 9.0 for Windows (StataCorp LP, College Station, Texas).

For BMI and blood pressure, we identified and excluded implausible values and outliers more than 3 standard deviations from the mean (<5% of values removed). Participants were excluded if they were younger than 30 years. Descriptive statistics were generated by community (intervention and control) and by households within those communities (intervention and control). Between-group comparisons were made with 1-way analyses of variance for continuous variables and analyses of proportions for noncontinuous variables.

We first examined the association between participation in *Oportunidades* and various adult health outcomes by conducting simple OLS regressions (linear and logistic) with program participation, age, and sex as independent variables. We then repeated these analyses with the 47 demographic, household-level, and community-level control variables described above. Standard errors were adjusted for intercluster correlation at the community level.

In order to examine the robustness and sensitivity of the OLS estimations, we also used nonparametric matching methods to assess the effect of *Oportunidades* on adult health. These methods are nonparametric techniques that control for observable differences between intervention and control communities. The methods can be more flexible than multiple regression methods, which rely strongly on assumptions of linearity (28). Because of limited space and the similarity of the outcomes with these 2 approaches, results reported are based on the standard OLS regressions.

### Ethical review

The *Oportunidades* evaluation was approved by the research committee at the National Institute of Public Heath in Mexico and the Committee for the Protection of Human Subjects at the University of California at Berkeley. Participants were invited to participate in the evaluation after receiving a detailed explanation of the survey procedures and were asked to sign an informed consent declaration at that time.

## Results

The *Oportunidades* and control communities were well-matched according to baseline (1997/1998) and current (2003) community-level variables (Table 1). The means of 19 out of 21 community variables were not significantly different from one another. Outcomes favored the control communities in terms of having received government assistance in 2003 and having higher female agricultural wages. We observed some differences between groups in household characteristics (Table 2); specifically, *Oportunidades* households owned more animals and small and large assets (including vehicles) and were less likely to have a dirt floor and more likely to have a bathroom.

### BMI and obesity

Age- and sex-adjusted BMI was significantly lower in the intervention group than in the comparison group (26.57 kg/m^2^ vs 27.16 kg/m^2^, *P* < .001), and the significance was attenuated with the inclusion of individual-, household-, and community-level covariates (Table 3). The prevalence of age- and sex-adjusted obesity was also significantly lower in the intervention group (20.28% vs 25.31%, *P* < .001) as was the prevalence of overweight (59.24% vs 63.04%, *P* = .03); the difference in obesity but not overweight was retained after adjusting for covariates.

### Hypertension

*Oportunidades* participants had lower SBP and DBP than did adults in comparison areas, when controlling for all covariates. Uncontrolled hypertension was present in 33.80% of the *Oportunidades* participants and 34.52% of the comparison group. Participation in the program was associated with increased likelihood of a participant's having had a blood pressure test in the 5 years before the survey, which was part of the *Oportunidades* program requirements. Program participation was also associated with a reduced number of self-reported symptoms relating to hypertension, such as headaches, dizziness, or buzzing in the ears.

### Self-reported health and behavior

The *Oportunidades* group was better able to participate in medium-effort ADL than was the control group, and the results were sustained after including covariates. Adults living in *Oportunidades* communities had fewer sick days in the 4 weeks before the survey and fewer days in which they were unable to do household chores. No significant difference was seen between the groups in self-reported ability to perform heavy-effort ADL.

## Discussion

Adults from households that had participated for 3.5 to 5 years in the large-scale incentive-based welfare program *Oportunidades* had a reduced prevalence of obesity and hypertension and better self-reported health than did adults from newly recruited control households. Most results remained significant after controlling for a large number of individual, family, and community characteristics.

The reported effects of the *Oportunidades* program on BMI and blood pressure are modest and of little clinical relevance for individuals (29), although they are likely to be important at the population level. Clinically significant weight loss has been defined as a loss of 5% to 10% of baseline weight (30) or a 4-pound minimum weight loss (31); our results show weight differences between the *Oportunidades* and control groups in BMI of only 2.2%. Our results show differences between groups for SBP and DBP of 1% to 3% (depending on covariates included). These findings are equivalent to or somewhat smaller in magnitude than results shown in a study of elderly Mexicans who were randomly assigned to receive biweekly or monthly home visits by a nurse for 6 months (32) and to results shown in an assessment of the effect on Brazilian adults of 3 individualized nutrition counseling sessions over the course of 6 months (33). Our results are much smaller in magnitude than are those of more intensive behavioral interventions or those that have used pharmacologic methods (34). In spite of the modest differences seen in blood pressure and BMI, however, differences of greater magnitude were evident when comparing the prevalence of obesity and hypertension. These results suggest that the differences in means between groups may have been less perceptible and were more likely to be seen at the higher end of the blood pressure or BMI distribution.

Despite our conservative analytical approach (we included almost 50 covariates in our adjusted statistical models), the major limitation of our analysis is that some household and community characteristics were not similar across groups. Given these differences, one of these variables may have contributed to the differences in outcomes observed in the intervention and control communities. However, we replicated our results with nonparametric techniques and feel confident that the analyses reported here allow us to control for a wide range of exogenous variables so that we can interpret our findings as effects of the program.

Another major limitation of the analysis is that we have baseline values for participants recruited in 1997 and 1998 but not for the comparison group recruited in 2003. Thus, we were forced to use self-report of retrospective data regarding household conditions 5 years before the current survey, which raises concerns about recall biases. However, we spent substantial energy and resources to pilot-test the questions used in the retrospective assessment so that the questions asked were either relatively constant over past 5 years or related to major changes of households; these strategies were designed to minimize recall errors.

The *Oportunidades* intervention is unique in that it combines 2 traditional types of interventions: cash transfer and direct provision of health care and services. *Oportunidades* increases purchasing power by permitting adults to choose what goods they want to buy and allows them choice about the quantity and quality of their purchases. Although participants may not understand the benefits of health-promoting behaviors and spend the money on other goods or services, 70% of the money appears to be spent on food (36). Given the design of *Oportunidades*, it is difficult to disentangle the causal pathways that contribute to the lower prevalence of obesity and hypertension. However, we speculate that the positive effects resulted from improved dietary quality, increased activity level, and increased monitoring of health outcomes.

*Oportunidades* has been shown to change patterns of food consumption in the short term (35). This pattern of intake goes against the trend in Mexico and Latin America, where intake of total fat, animal products, and sugar are increasing at the same time as consumption of cereals, fruit, vegetables, and traditional diets are decreasing (36). One reason that these rates of consumption of calorie-rich foods have increased on a national level could be that the costs of consuming fats and sugar have gone down. In spite of the increased ability of *Oportunidades* participants to afford more fat and sugar because of their increased income, the lower prevalence of obesity and hypertension in the intervention areas suggests that the educational component of the program could be counterbalancing the income effect.

Another explanation for our findings is that adults in *Oportunidades* communities may have been more physically active. Our results suggest that people in those areas had higher activity levels than did those in comparison communities, which could have contributed to weight loss and better overall health. Previous research conducted in Mexico has shown low levels of physical activity in Mexican women (37), particularly women with only a primary school education. Thus, even a minimal increase in physical activity could decrease BMI.

A third potential explanation for our findings is that adults are being checked more regularly for health outcomes as a result of the *Oportunidades* program requirements. Specifically, participants must have a health checkup twice yearly and must participate in regular health promotion sessions in order to receive program benefits. Our findings suggest that the program increased doctor visits by the participants, particularly given the higher rates of uncontrolled hypertension in *Oportunidades* households. The doctor visits may also have increased access to information about the risks of overweight, obesity, and hypertension and may also have had a positive effect on household and social norms about food intake and activity.

Participation in *Oportunidades*, a wide-reaching poverty alleviation program, was associated with small but significant reductions in BMI and blood pressure and the prevalence of obesity and hypertension and improved self-reported health outcomes. Although *Oportunidades* was designed to target and improve health and development in children, it also appears to have improved the health of adults. Although the clinical significance of the BMI and blood pressure findings is minimal, the effect at the population level may be meaningful, particularly given the rapidly increasing prevalence of chronic diseases in Mexico. Other large-scale interventions have shown that even small findings at an individual level translate to larger effects at the community level (38,39). The findings suggest that large-scale poverty alleviation initiatives paired with health behavior requirements may be a first step toward curbing the rapidly increasing prevalence of obesity and related noncommunicable diseases around the world.

## Figures and Tables

**Table 1:** Baseline Characteristics of Rural, Low-Income *Oportunidades* Communities (1997/1998) and Control Communities (2003), Mexico

Characteristic	*Oportunidades* (n = 264)	Control (n = 59)	*P* Value^a^
Some indigenous population in the community, %	33.48	28.86	.65
At least some part of the village supplied with electricity, %	74.12	68.86	.78
At least one private or public telephone in 2003, %	81.38	72.53	.10
At least one preschool before 1998, %	88.90	80.10	.07
At least one primary school before 1998, %	90.36	92.49	.66
At least one health center before 1998, %	33.81	37.72	.91
Had local shop in 2003, %	24.76	24.83	.83
Had home-based shop in 2003, %	60.50	61.97	.95
Community received *desayuno* school food program in 2003, %	72.46	77.54	.35
Community received *despensas*, food stamp assistance in 2003, %	41.64	59.42	.02
Mean distance to distance learning center, km (SD)	2.8 (3.6)	3.0 (3.0)	.65
Distance learning center in community, %	24.00	17.73	.19
Mean seasonal rent, pesos (SD)	96.52 (218.93)	94.26 (208.77)	.94
Mean cost to rent irrigation equipment, pesos (SD)	51.17 (253.04)	48.61 (220.71)	.94
**Mean wage, pesos (SD)^b^**			
Male agricultural worker	880.31 (595.78)	1099.25 (888.17)	.07
Female agricultural worker	345.15 (418.55)	561.30 (521.87)	.003
Child agricultural worker	203.04 (302.80)	232.65 (340.18)	.53
Male nonagricultural worker	393.47 (1188.64)	710.66 (1620.87)	.15
Female nonagricultural worker	161.52 (1055.52)	204.67 (461.82)	.63
Male employed worker	312.81 (1581.80)	201.02 (472.16)	.33
Female employed worker	175.93 (1047.41)	379.74 (1839.89)	.41

a Differences between groups assessed by using tests of means or tests of proportions, adjusted for clustering at the community level.

b All wages are monthly and from 2003, deflated to 1997 levels. US$1 = approximately 10 Mexican pesos. "Nonagricultural workers" are informally employed and paid, whereas "employed workers" are formally employed and salary-based.

**Table 2:** Baseline Individual and Household Characteristics of Rural, Low-Income *Oportunidades* Participants (1997/1998) and Controls (2003), Mexico

Characteristic	*Oportunidades* Households (n = 5280)	Control Households (n = 1063)	*P* Value^a^
**Individual characteristics**			
Mean age, y, in 2003 (SD)	41.87 (8.88)	41.75 (9.01)	.76
Female, %	68.04	71.02	.42
No primary education, %	25.20	22.69	.82
Married, %	69.77	74.60	.20
Height, cm, mean (SD)	152.00 (8.59)	151.93 (8.25)	.91
**HOH characteristics**			
Speaks indigenous language, %	41.23	30.28	.34
Education, y, mean (SD)	3.47 (2.86)	3.47 (2.85)	.97
Spouse education, y, mean (SD)	3.26 (2.72)	3.31 (2.77)	.85
**Household characteristics**			
No. of people in household, mean (SD)	6.35 (2.05)	6.08 (2.27)	.17
No. of children aged <5 y, mean (SD)	1.19 (0.97)	1.29 (1.08)	.25
No. of working members of household, mean (SD)	1.63 (1.02)	1.75 (1.22)	.14
No. of disabled members of household, mean (SD)	0.04 (0.18)	0.05 (0.20)	.47
No. of people aged >55 years, mean (SD)	0.30 (0.55)	0.27 (0.57)	.31
Crowding, no. of people/room, mean (SD)	4.31 (2.09)	4.40 (2.13)	.60
**Housing and assets**			
Land owned, hectares, mean (SD)	2.16 (2.99)	2.49 (5.05)	.67
Farm animals owned (1 = cow-equivalent), mean (SD)	0.39 (1.16)	0.23 (0.77)	.002
Other animals owned (1 = cow-equivalent), mean (SD)	1.43 (2.80)	1.00 (3.36)	.01
Have dirt floor, %	63.87	71.48	<.001
Have bathroom, %	61.18	57.19	<.001
Have electricity, %	77.20	77.52	.96
No. of large assets, mean (SD)^b^	0.97 (0.99)	0.63 (0.89)	.002
No. of small assets, mean (SD)^c^	1.17 (0.90)	0.83 (0.90)	<.001
Own vehicle, %	5.47	2.05	<.001
No. of rooms in household, mean (SD)	1.80 (0.99)	1.67 (1.15)	.06
Roof is concrete or other durable material, %	13.18	14.70	.76
Walls are concrete or other durable material, %	49.01	57.67	.34

HOH indicates head of household; if the HOH was not available, the spouse of the HOH was interviewed.

a Differences between groups assessed by using tests of means or tests of proportions, adjusted for clustering at the community level.

b Such as television, washer, gas heater, and refrigerator.

c Such as blender, electric kettle, radio, stereo, video cassette recorder, and fan.

**Table 3:** Differences Between Rural, Low-Income Adults in *Oportunidades* Communities and Those in Control Communities, Mexico

Characteristic	*Oportunidades* (n = 5280)	Control (n = 1063)	*P* Value^a^	Adjusted Effect (β)^b^ (95% CI)	*P* Value for β
**Body mass**					
BMI (kg/m^2^), mean (SD)	26.57 (4.62)	27.16 (4.79)	<.001	–0.47 (–0.97 to 0.03)	.07
% obese (BMI ≥30.0 kg/m^2^)	20.28	25.31	<.001	–0.04 (–0.08 to 0.00)	.03
% overweight (BMI ≥25.0 kg/m^2^)	59.24	63.04	.03	–0.02 (–0.07 to 0.04)	.51
**Blood pressure and heart rate**					
Systolic blood pressure, mm Hg, mean (SD)	123.73 (16.56)	124.47 (15.95)	.11	–2.60 (–4.09 to –1.11)	<.001
Diastolic blood pressure, mm Hg, mean (SD)	81.55 (13.17)	82.41 (13.54)	.03	–2.84 (–4.38 to –1.30)	<.001
Heart rate, beats per minute, mean (SD)	76.27 (9.31)	76.80 (9.40)	.10	–0.88 (–1.93 to 0.18)	.18
Uncontrolled hypertension, %^c^	33.80	34.52	.48	–0.07 (–0.12 to –0.02)	.008
Blood pressure checked within5 years before survey, %	87.60	79.87	<.001	0.06 (0.02 to 0.10)	.004
No. of self-reported hypertension-related symptoms, mean (SD)^d^	1.43 (1.37)	1.71 (1.42)	<.001	–0.24 (–0.37 to –0.11)	<.001
**Self-reported health**					
Medium-effort ADL, %^e^	94.66	92.32	.005	0.03 (0.01 to 0.05)	.03
Heavy-effort ADL, %^f^	92.11	91.58	.63	0.01 (–0.01 to 0.04)	.31
No. of sick days in past 4 weeks (SD)	3.42 (6.81)	4.23 (7.45)	<.001	–0.48 (–1.16 to 0.19)	.16
No. of days unable to do ADL (SD)	1.03 (3.42)	1.52 (4.06)	<.001	–0.55 (–0.85 to –0.25)	<.001

CI indicates confidence interval; BMI, body mass index; ADL, activities of daily living.

a *P* value reported for differences between means or proportions generated with regressions controlling for age and sex, clustered at the community level.

b Random effects regression was used, clustering at the community level. Sample size was within 50 observations of n = 5280 adults in *Oportunidades* communities and n = 1063 adults in comparison communities, although actual number varies by dependent variable. Regressions were adjusted for more than 50 variables (those listed in Tables 1 and 2), including individual-level variables (e.g., age, sex, education, marital status), household-level variables (e.g., household size, demographic structure, economic status), and community-level variables (e.g., proportion of community with indigenous population, presence of electricity in community).

c Systolic blood pressure ≥140 mm Hg or diastolic blood pressure ≥90 mm Hg.

d Headaches, dizziness, buzzing in the ears, seeing lights without apparent reason, nosebleeds without apparent reason.

e Able to work on a farm, work in a garden, or sweep.

f Able to run or lift a heavy object.
